# Introducing CRAFT: The Center for Research and Advancement in Fragments and molecular Targets

**DOI:** 10.1021/acsmedchemlett.4c00296

**Published:** 2024-07-23

**Authors:** Carolina Horta Andrade, Maria Cristina Nonato, Flavio da Silva Emery

**Affiliations:** † Center for Research and Advancement in Fragments and Molecular Targets (CRAFT), School of Pharmaceutical Sciences at Ribeirao Preto, University of São Paulo, Ribeirão Preto, São Paulo 05508-060, Brazil; ‡ Laboratory for Molecular Modeling and Drug Design (LabMol), Faculty of Pharmacy, Universidade Federal de Goiás, Goiânia, Goiás 74605-170, Brazil; § Center for Excellence in Artificial Intelligence (CEIA), Institute of Informatics, Universidade Federal de Goiás, Goiânia, Goiás 74605-170, Brazil; ∥ Protein Crystallography Laboratory, Department of Biomolecular Sciences, School of Pharmaceutical Sciences at Ribeirao Preto, University of São Paulo, Ribeirão Preto, São Paulo 05508-060, Brazil

**Keywords:** fragments, artificial intelligence, molecular targets, neglected diseases, fragment-based drug discovery

## Abstract

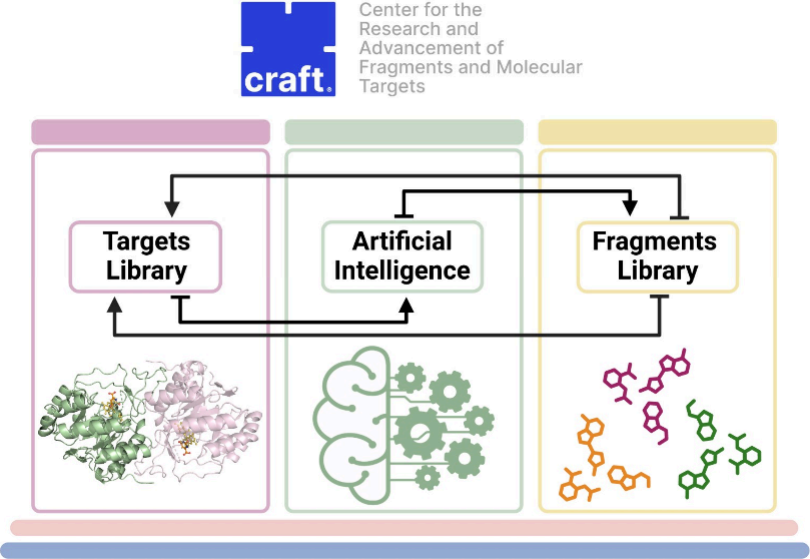

We introduce the Center for Research and Advancement in Fragments and Molecular Targets (CRAFT), a pioneering research center established in 2021 through a collaboration between the University of São Paulo (USP) and the Federal University of Goiás (UFG). CRAFT integrates fragment-based drug discovery (FBDD), artificial intelligence (AI), and structural biology to develop novel therapeutic strategies. We have created fragment and target libraries and utilize AI models to streamline the drug discovery process. We invite the global scientific community to collaborate with us in addressing neglected diseases, with the goal of enhancing research capabilities and fostering scientific innovation across Latin America.

According to the Global Health Estimates (2019), infectious diseases, including parasitic and lower respiratory diseases, accounted for approximately 14% of global deaths.^1^ This alarming statistic was significantly influenced by COVID-19, particularly in sub-Saharan Africa, the Middle East, South Asia, and Latin America, where the burden of excess deaths and all-age excess mortality rates exceeded the global average.^2^ It is no coincidence that these same regions bear the brunt of Neglected Tropical Diseases (NTDs). A combination of factors including low social demographic indices, inadequate public policies, underdeveloped health sectors, insufficient investments in science and technology, environmental degradation, and climate change contribute to the high prevalence of NTD-related disabilities in low- and middle-income countries.^3^

Interestingly, in 2023 there was a notable surge in newly FDA-approved entities, marking a resurgence in both anti-infective small molecules and biologic products. Out of the 63 FDA-approved new entities, 12 were introduced to address a diverse range of infections. These include Nirsevimab, an inhibitor of the Respiratory Syncytial Virus (RSV) F protein, Nirmaltrevir (Paxlovid), an inhibitor of the SARS-CoV-2 main protease, and Durlobactam, a beta-lactamase inhibitor used in the treatment of bacterial pneumonia. The total number of FDA-approved drugs reached one of the highest levels seen in recent decades, with anti-infective therapies and vaccines comprising 15% of the total approvals. The drug discovery process for the four anti-infective small molecules approved last year involved a variety of approaches, including compounds inspired by natural products such as Taurolidine, used for catheter-related bloodstream infections, and optimized echinocandins like Rezafungin to treat candidaemia and invasive candidiasis.

These recent approvals have underscored the increasing clinical impact of structure-based drug discovery (SBDD) in combating infections. However, while the fragment-based drug discovery (FBDD) approach has become a well-established strategy for accelerating the discovery of clinical candidates, its application in the development of antimicrobials has proven challenging. Most of the fragment-based discovered drugs were developed for cancer, with few others developed for neurologic disorders, inflammation, autoimmune diseases, and other conditions. To our knowledge, only one example of fragment-based antimicrobial compound has advanced to clinical trials, albeit without demonstrating efficacy. This compound, AZD5099, was derived from a pyrrole ethyl ester fragment and aimed to target the bacterial ATP-competitive GyrB/ParE subunits of type II topoisomerase.^4^ It exhibited significant activity in animal models of methicillin-resistant *Staphylococcus aureus* (MRSA) infections. However, due to toxicity and safety concerns, the clinical trial was halted.

The significant societal and global health burden posed by infectious diseases, coupled with the scientific complexities inherent in drug discovery, served as the driving force behind the establishment of the Center for Research and Advancements in Fragments and Molecular Targets (CRAFT). CRAFT is an academic interinstitutional Brazilian research center connecting the University of Sao Paulo (USP) and the Federal University of Goias (UFG). The journey toward CRAFT began in 2013,^5^ when the teams led by Professors Nonato and Emery at USP embarked on a fruitful collaboration aimed at comprehending the structural and functional characteristics of the enzyme dihydroorotate dehydrogenase (DHODH) across various species. This effort utilized diverse biophysical chemical tools, leading to the discovery of nanomolar inhibitors for DHODH in different species.^6−9^

This collaboration reached its pinnacle with the approval of a grant from the National Institutes of Health (NIH – 1R01AI160379-01), which provided the team with the resources to consolidate efforts and infrastructure into a centralized structure. The success of this endeavor facilitated the expansion of activities, culminating in the integration of AI expert Professor Andrade from UFG into the team, transforming it into a multicenter platform, and allowing the approval of multinational grant within BRICS STI Framework Programme (grant 441038/2020-4).

With our organization established, CRAFT’s vision, mission, and goals have become distinctly clear, particularly given our background in the research on Brazilian endemic diseases. CRAFT’s mission is centered on advancing Brazilian Drug Discovery, educating future leaders in the field, and fostering citizen engagement to work together with the community in public health issues. These objectives are underpinned by a set of core values that cultivate an environment rich in citizenship, collaboration, curiosity, ethics, and scientific engagement.

We stress the utmost importance of directing research endeavors toward the discovery of therapeutics for infectious diseases, particularly those that disproportionately impact impoverished and developing nations. CRAFT places considerable emphasis on investigating neglected infectious diseases, bacterial and viral diseases. CRAFT is also devoted to pandemic preparedness, investing in research for developing therapeutics for emerging pathogens, including Mayaro, Marbourg and Oropouche viruses, among others.

Scientifically, the center is pioneering in integrating AI to FBDD for infectious diseases, a cutting-edge approach in the development of therapeutics for global health needs.

We are deeply engaged in the exploration of fragment screening techniques across a spectrum of methodologies to identify ligands for a diverse range of pharmacological targets. CRAFT’s portfolio encompasses three different pillars: development of fragment libraries; structural biology focused on protein targets; and Artificial Intelligence (AI) models development and computer-assisted tools to drive the drug discovery campaigns. Each of the pillars are discussed below.

One of CRAFT’s pillars is the development of an innovative library of fragments based on new heterocyclic scaffolds and natural product derived compounds. Our group has been developing fused heterocyclic systems that have either not been synthesized before or are underexplored in medicinal chemistry.^10−12^ These new fragments pose synthetic challenges that our group has successfully tackled and are pivotal in overcoming intellectual property barriers, thus driving innovation. More interestingly, we expand the library of these new heterocycles by studying the vectorial functionalization of these cores, to make useful compounds for FBDD. For this, we use state-of-the-art functionalization strategies, such as CH-activation, photocatalyzed reactions, and radical-based strategies for achieving unusual positions at the ring, in order to get a diverse library in terms of types of functional groups and vectors explored.

Furthermore, we are developing natural-product based compounds to expand our library of fragments. In this case, two types of approaches have been developed—fragment-like natural products and natural product derived fragments. For the first case, we explore libraries of Brazilian natural products available, do the total synthesis of heterocyclic natural products, and explore further functionalization and synthesis of analogues.^13^ For the library of natural product derived fragments, we decompose complex natural products into smaller fragments, trying to expand the chemical space of tractable compounds, while escaping from the flatland of fused aromatic heterocycles. These compounds are incorporated into CRAFT’s library and used in our screening platform.

The second pillar of CRAFT is the target library. Here, we have cultivated expertise in heterologous production and characterization of target proteins,^14−16^ including the international recognition for our extensive work on the drug target dihydroorotate dehydrogenase.^17^ This encompasses not only biochemical, biophysical, and structural characterization, which provide essential insights for mapping function, but also the development of suitable fragment screening protocols. We utilize enzymatic kinetic assays, differential scanning fluorimetry (DSF), and many other biophysical techniques, selecting the appropriate ones based on the specific problem at hand.

With our strong foundation in X-ray crystallography on single crystals, CRAFT is spearheading the application of fragment screening via X-ray crystallography in Brazil. Moreover, X-ray crystallography plays a pivotal role in elucidating the structural foundations of protein–ligand interactions, thus guiding the design of next-generation ligands.

The third pillar of CRAFT is the development and application of AI and machine learning (ML) models as well as structure-based drug design (SBDD) and ligand-based drug design (LBDD) strategies for hit identification and hit-to-lead optimization in both target-based^18,19^ and phenotypic-based approaches.^20−22^ We also explore a range of *in silico* methods to facilitate the subsequent optimization of fragments into lead compounds through growth, linking, and merging. These strategies for expanding fragments encompass hot spot analysis, druggability prediction, SAR (structure–activity relationships) analysis using catalog methods, the application of ML-QSAR models for virtual screening, and generative design for proposing synthesizable new compounds. We also develop AI and QSAR/QSPR models for toxicological research and risk assessment of chemicals.

The integrative approach of CRAFT can streamline the preclinical drug discovery pipeline, from the initial screening of fragment libraries to the optimization of lead compounds. Figure 1 illustrates the workflow of how the three pillars of CRAFT operate.

**Figure 1:**
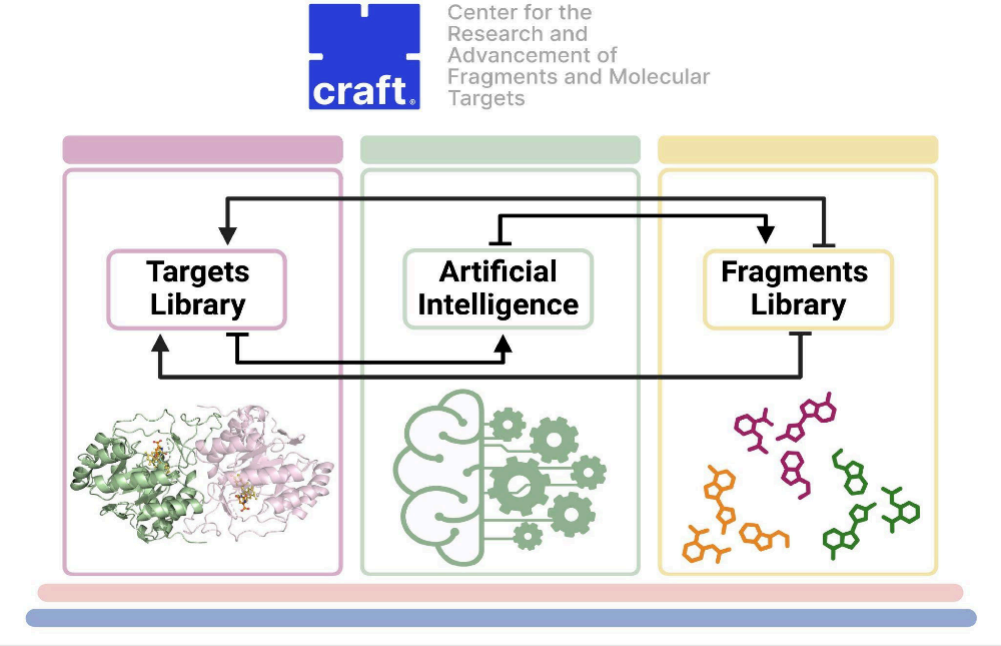
Integrative approach of CRAFT highlighting its three pillars: fragment library, targets library and AI approaches.

CRAFT aims to establish itself as a global leader in the quest for treatments for endemic diseases prevalent in Latin America and the Global South, advancing drug discovery in Brazil and throughout Latin America. Our recent initiatives include the partnership with the Welcome Centre for Anti-Infectives Research (WCAIR) at the Drug Discovery Unit (DDU) of the University of Dundee. This collaboration brought the “Drug Discovery Mission” short course to the Brazilian community, providing hands-on experience in decision making for the selection and evolution of hits toward clinical candidates. Additionally, in partnership with DDU, we co-organized a workshop on Drug Metabolism and Pharmacokinetics (DMPK) to integrate the academic and industrial medicinal chemists with Brazilian pharmacokinetics experts. This two-day workshop tackled a critical area that is underdeveloped in Brazil, hindering innovation in drug discovery. Strategies to optimize resources and funding for Pharmacokinetic-Pharmacodynamic (PKPD) groups facilities were discussed. To further this effort, we are developing the fourth pillar of CRAFT: an *in vitro* PKPD facility.

Finally, we are working on establishing a Latin America Drug Discovery Network as part of a recently approved grant (CNPq - 443750/2023-8). This network aims to connect groups, expand capabilities, and increase scientific and technological autonomy in developing regions, reducing dependency on APIs and innovative drugs from major pharmaceutical companies.

CRAFT is also committed to fostering an inclusive environment for new researchers, including students and postdocs, who are interested in studying and working in the field of drug discovery and development with us. We also welcome new partners to collaborate on challenging projects and drive innovation together.

In summary, CRAFT is a manifestation of collaboration, scientific curiosity, and citizenship, to shape the Brazilian and Global South health needs through innovative drug discovery, driven by the ethical imperative to deliver therapeutics that serve the public good.

## ABBREVIATIONS

COVID-19: coronavirus disease 2019
NTD: neglected tropical diseases
FDA: Food and Drug Administration
RSV: respiratory syncytial virus
SARS-CoV-2: Severe Acute Respiratory Syndrome Coronavirus 2
SBDD: structure-based drug discovery
FBDD: fragment-based drug discovery
GyrB/ParE: DNA Gyrase Subunit B and Topoisomerase IV Subunit E
MRSA: methicillin-resistant *Staphylococcus aureus*
CRAFT: Center for Research and Advancements in Fragments and Molecular Targets
USP: University of Sao Paulo
UFG: Federal University of Goias
DHODH: dihydroorotate dehydrogenase
NIH: National Institutes of Health
BRICS STI: Brazil, Russia, India, China, and South Africa - Science, Technology, and Innovation
AI: Artificial intelligence
DSF: Differential Scanning Fluorimetry
ML: machine learning
LBDD: ligand-based drug discovery
SAR: structure–activity relationship
ML-QSAR: QSAR/QSPR, Quantitative Structure–Activity Relationship/Quantitative Structure–property Relationship
WCAIR: Welcome Centre for Anti-Infectives Research
DDU: Drug Discovery Unit
DMPK: Drug Metabolism and Pharmacokinetics
PKPD: Pharmacokinetic-Pharmacodynamic
CNPq: National Council for Scientific and Technological Development
